# Farinaceous Infant Foods

**Published:** 1882-07

**Authors:** 


### ARTICLE VIII.

### *Farinaceous Infant Foods.*

---

Under the above title Dr. Geo. B. Fowler publishes in *The American Journal of Obstetrics and Diseases of Women and Children*, Vol. xv., No. 2, April, 1882, an inquiry into the chemical composition of the principal prepared foods for infants now found in the market. Dr. Fowler states that he was impelled to undertake the critical examination of some of these preparations on account of the importance of discovering as perfect a substitute as possible for mother's milk, because of the frequency with which we are compelled to employ these foods, and on account of the conflicting experience of the profession regarding their relative utility. He specially emphasizes the fact "that simple microscopic inspection, unaided by chemical means and physical processes, is wholly unreliable and inadequate in determining the composition and nutritive worth of these farinaceous compounds." Dr. Fowler begins the discussion of his subject by alluding to the relative nutritive value of the various grains employed in the preparation of the foods in question, with a view to forming an approximate estimate of relative proportions in which each should be employed. This basis for a scientific discussion of the subject is founded upon the important physiological fact that, to fully subserve the purposes of natural nutrition, foods must contain albuminoids, hydrocarbons, and mineral matters in proper proportion. Since the proportion in human milk, the natural food for infants, between the albuminoid and non-albuminoid ingredients is as 1 to 2.95, it is just to assume that the same relations should obtain in the artificially prepared aliment. These data would seem to justify our belief that the most desirable substitute for human milk may eventually be synthetically ompounded. No elementary food product, however asily digested when employed alone, is sufficient for the maintenance of health or even of life. By proper processes it is, however, possible to vary the relative proportions of constituents in flour produced from any grain by the admixture of flour from other cereals. In this way a food sufficient for prolonged normal nutrition may be prepared. Dr. Fowler next formulates the leading facts relating to the structure of different cereal seeds, and states the chief microscopical features of gluten, starch, dextrine, and other ingredients of foods obtained from this source. The author then gives the results obtained by chemical microscopical examinations of the various foods of the shops. Their names are mostly familiar. The chief ones are Horlick's Food; Imperial Granum; Ridge's Food; Nestle's Milk Food; Anglo-Swiss Milk Food. Dr. Fowler shows that the actual composition of many of these preparations does not at all correspond with the published statements concerning them. He does not, however, attempt to decide which of the foods is best adapted to the wants of infants, but leaves this question to be settled by practical experiments based upon chemical and microscopical examinations such as he has conducted.—*Med. Record.*

---

